# Opium-smoking

**Published:** 1886-02

**Authors:** Chas. Warrington Earle

**Affiliations:** Physician to the Washingtonian Home


### OPIUM-SMOKING IN CHICAGO. *By* CHAS. WARRINGTON EARLE, M. D., *Physician to the Washingtonian Home.*

[Read before the Chicago Medical Society, January 4th, 1886.]

The vices of the old world rapidly find their way to our shores, and along with alcoholic excesses, and opium-eating, and the cocaine habit, we find ourselves confronted by a new and, in some respects, a more fascinating vice, opium-smoking.

The narcotic properties of the juice of the poppy were known two or three centuries before the commencement of the Christian era. I am, however, without the proper data as to the time when crude opium, morphine, codeia, etc., etc., were first separated from it.

The practice of eating and smoking opium probably was introduced into China from Arabia. From China it was brought to California, and what we know of the bad effects of the drug has come from our western shores. Until the year 1868, opium-eating and opium-smoking were practiced and indulged in only by the Chinese. The first white man, a sporting character, smoked opium in California (1868). This, then, is the starting point in this country.

I find in looking over the reports, and particularly an article entitled "Number of Opium-smokers in China," from the *North China Daily News*, that it is estimated that about two millions of Chinese are opium-smokers, and although this evil is apparently so general, there is also a deep feeling among this numerous people that its use is doing a widespread and irreparable injury to their country. These people have come to this country bringing the habit with them, and in their own apartments and in sections of cities set apart for their habitation, they carry on the vice, as they learned it and practiced it in their native land. Two millions of inhabitants of a country indulging in a habit of this kind seems terrible. It is terrible, but not to the extent which the figures indicate, for it is only two-thirds of one per cent. of the inhabitants—as the estimate of the population is 400,000,000.

A certain number of young men in our own country, Americans, with nothing to do, prostitutes, and young men and girls willing to try some experiment to make life somewhat more fascinating; or that class of people who appear to have no aim in life except to gratify some abnormal appetite, have come to learn and practice this habit.

My experience is with young men all Americans, I think, and all with capabilities which, if exerted in the right direction, would have enabled them to occupy a fine and, in one or two cases, more than an average position among their associates. They lacked early discipline, however, and contracted this with two or three other pernicious habits.

It was soon noticed by the authorities in California that the practice was extending, and efforts have been made to prohibit by law the spread of this vice. In California a law exists making every person who opens an establishment where opium-smoking may be carried on, or who resorts to these places, punishable either by fine or imprisonment. In Nevada, also, laws have been passed making this vice punishable by sentence to the penitentiary; and it is believed by the authorities that the evil habit has been in a very great degree checked by these very stringent laws. In our own city, during the last three or four years, the police have made various efforts to break up certain smoking dens, and have been in a very great degree successful. I am not prepared to say that much of this smoking is not now carried on in private apartments. The Chinese smoking-dens have been almost entirely destroyed.

A smoker's outfit, which I now show you, is somewhat expensive, and the practice itself is one which costs a considerable amount of money. The pipe is from 16 to 24 inches in length, and consists of a bowl and stem. In addition there is a large tray, upon which is placed a lamp, several peculiar shaped knives and scissors for the cleansing of the pipe, and for the trimming of the lamp; a long needle, and a peculiar shaped box or can in which the opium is placed. The place where the opium-smoking is usually carried on is called an "opium-joint," and in this city we very frequently find the front part of one of these "joints" occupied as a cigar store or a laundry, the "joint" being well towards the rear of the establishment. If one is well fitted up there are several bunks or low tables, upon which are placed certain kinds of matting. One of my informants has seen eighteen people, male and female, smoking in one of these dens at one time.

As I remarked above, a place where opium-smoking is carried on is called an "opium-joint." One who is thoroughly under the influence and *must* smoke frequently is called a "fiend." The act of smoking with all its preparations is termed, "hitting the pipe."

The first "opium-joint" I ever visited was upon State street. A young woman had become addicted to this habit; and a gentleman, somewhat interested in her, appealed to me to visit her and if possible prevail upon her to place herself under restraint. I found the patient in one of those low wooden buildings on South State, near 16th street. A small cigar store occupied the front of the building, the second room being filled with gaudily dressed girls, more particularly of French descent, while back of this was a fairly furnished parlor, into which I was ushered. In the course of my conversation with this young woman and the gentleman who was interested in her, I requested to see the outfit for smoking, and if not particularly objectionable, to see them smoke. They very readily acquiesced, and repaired at once to an inner room, and went through the entire process of cooking and smoking opium. A low bed, rather luxuriously furnished, occupied one side of the room, upon which the gentleman placed the tray containing the outfit. Reclining on the couch, one on either side of the tray, they proceeded to cook and smoke the opium. My efforts at reforming this woman were not at all successful, and she declined placing herself under the treatment which I suggested. She died some months after in Cook County Hospital. My attention has, more particularly, been called to this vice by the following histories of men, who have become inmates of the Washingtonian Home during the last two years. Their ages, nationalities, occupations, etc., are briefly given.

No. 1. Age, 38; born in Tennessee, gambler by occupation, smoked for the relief of neuralgia since 1880. Was admitted on pretence of desiring to reform from alcoholic excesses, but in a few days confessed that he was an opium-smoker, and was placed under the usual treatment. In five days he was perfectly free from his opium-habit, and sent to the other side of the Home.

No. 2. Age, 28; born in Louisiana, bar-tender, commenced smoking in 1880. Was in the hospital six days when he was sent to the reformatory side of the house. He was discharged some days after for violating the rules of the institution, and is now tending bar and probably smoking.

No. 3. Age, 27; born in New York, son of a wealthy man and with nothing to do; smoked since 1880. While under treatment did excellently well, and was sent to the reformatory with every prospect of a perfect reformation. He is known now to frequent a saloon whose attachés are opium-smokers, and presumably he is smoking.

No. 4. Age, 25; born in New York, telegraph operator; smoked opium since 1881. Several days after admission he was discharged from the institution for bringing whisky into the house.

No. 5. Age, 28; born in Pennsylvania, clerk; commenced the habit in San Francisco in 1878. Did well for several days, but finally was discharged on account of drinking.

No. 6. Age 26; American, telegraph operator; married. Up to seven years ago he was addicted to no habit. At that time he met with an accident, for which his physician prescribed morphine. During the year after his discharge by the physician he took the drug until he was using five grains daily. Six years ago he began smoking opium as a substitute for the morphine habit. He is now under treatment and seems determined to make a success. His wife, from constant attendance upon him and from assisting him in preparing his pipe, has become an *habitué*.

OPIUM-SMOKERS IN CHICAGO.

The exaggerated statements made by some of the victims in regard to the number of those practicing this habit, should be taken with the greatest possible allowance. Several years ago I made a very careful investigation in regard to the number of *opium-eaters* in this city, and 2,000 would be an outside number, and yet, within a short time, I have seen a statement in the public prints, telegraphed from New York, that over 25,000, including a large number of the better class of our people, fashionable ladies and the like, were using this drug.

There are no facts which warrant such statements. In regard to those smoking opium the number is very much less. One of my informants, who is well acquainted with all of the smoking-dens, and with a great part of those addicted to the habit, believes that 60 will cover all who are known to be opium-smokers in this city, and I have never had an estimate of over 200 made by those who are in the habit of exaggerating to the utmost. We must always take with great allowance any estimate made by the *habitué*, for inasmuch as he uses the drug himself he is convinced that everybody must also use it. This peculiarity is true of many drunkards. Concerning the class of people who smoke opium, we find that they belong to that crowd of people who are easily tempted, with very little to do, usually associated with some saloon-interest, prostitutes, and occasionally one who has failed in every kind of business, and seeks oblivion by the use of the pipe. We have not been able to find that any of the respectable class of females are in the habit of using the pipe, and the stories that wealthy ladies are driven to these dens in their carriages and seek solace and become fascinated by the use of this drug are absolutely false. I know of but two ladies who use the pipe. Coming now to speak of the symptoms produced by smoking this drug, we find that they may be fairly divided into two classes: Symptoms experienced by the new beginner of an exhilarating and fascinating character. 2d, The symptoms experienced by the "opium-fiend," or one addicted to the use of it in quantities, and who takes it for the obtunding of all his sensibilities. Concerning the effects of the first, I will quote from a writing of a physician, who in order to be a better judge of the effects of the drug, had every facility for smoking opium erected in his own office,—a most dangerous experiment it seems to me. He says: "The first effect was nausea and dizziness, accompanied by a pleasant sense of exhilaration, and followed by a quiet easy contentment. After a time there was an increase in the force and frequency of the pulse, giddiness, with slight nausea, and some staggering on attempting to rise or walk. There was a feeling of uncertainty in putting down the feet in walking, dazing of the mind, sleepiness, and heaviness of the eyelids. There seemed to be some trouble with the ears, and he commenced talking very loud." At first the pulse, temperature, and respiration are slightly increased, but after the drug has been smoked some time, they are lessened. During the commencing stage every one is quite certain he can give up the use of the drug at any time, but as time goes on he finds himself completely under its influence, enslaved to a demon whose power he cannot possibly resist. The quantity used as compared with a given quantity of pulvis opii. or morphia I am not able to ascertain. It is a kind of watery extract and looks something like a thick black molasses. Its effects are very similar, though less intense, to taking morphine by the mouth, and, after a very careful consideration of the subject, I have come to the conclusion that smoking opium produces less injury to the system than taking it by mouth. The cure for opium-smoking is much easier, and the patient experiences much less pain than when recovering from its use when taken by mouth or hypodermically. Opium-smokers do not have that haggard, pale look that we almost always find in opium-eaters. A man may smoke opium in a moderate degree for years, and not injure his physical condition; but mentally and morally it produces the most terrible results. He becomes melancholy and sees things in a way that casts a gloom over all his prospects. He becomes indifferent to business, careless in regard to all the responsibilities of life, and above all there is a tendency to prevaricate and falsify, sometimes when there is absolutely no reason for so doing. Respiration and circulation, as I have already stated, are slightly increased during the first stage, but lessened when the person becomes thoroughly under the influence of the drug. There appears to be, when the *habitué* becomes entirely under the influence of the drug, a lessening in regard to the sexual appetite, but seminal emissions are frequent. During the interval of smoking or during the slight period of exhilaration, the sexual appetite is increased, and taking knowledge of this fact, it is claimed that a certain number of girls have been ruined by those knowing the peculiar desires and inclinations when partially under the influence of the drug. The use of the drug by smoking produces constipation and hæmorrhoids, the same as we find when taken by the mouth, or hypodermically, although all the symptoms are less intense. Upon its withdrawal we find sneezing, running at the eyes, dizziness, noises in the head, diarrhœa, muscular pains and insomnia present in a large number of cases. There is, however, but little suffering experienced by one recovering from this vice, compared with the sufferings endured by one who has been an opium-eater. Little medication is needed, but these persons must be under restraint, and must be under the control of one who is firm, and who under no circumstances will believe what they say. A few days in a room well warmed, that has all the conveniences of bathing, with cocoa, and avena sativa—nerve sedatives—and tonics, with a fluid diet, will bring a person to where he can commence to build up his mental and moral status. He is now in a condition in which he may commence to reform if he so desires. I have never yet met with a case which I can say was cured, or with a case that I believed was hopeless, or one who, I believed, could not reform from opium-smoking if he desired. These people will not accept the conditions necessary for a cure. The great trouble is that they are so oblivious to all the responsibilities of life, and so willing to become wrapped up with the exhilarating effect of this drug, avoiding all the troubles, pains, and responsibilities of life, that they cannot be brought to see that it is a duty both to themselves and to their friends to stop the habit. In addition we find a certain class of people who are willing to coddle, and treat these men and women for a disease; are willing to excuse them for the terrible train of symptoms which they have brought upon themselves, and which they insist upon retaining. In this way we are thwarted from bringing about good results, which might be produced.

